# Prognostic value of MRI T2 quantification in heart transplant patients: a 5-year outcome study

**DOI:** 10.1186/1532-429X-18-S1-O34

**Published:** 2016-01-27

**Authors:** Varun Chowdhary, Spencer Barfuss, Yi Gao, Julie Blaisdell, Mary Ackermann, Clyde Yancy, Allen Anderson, Jeremy Collins, Ann B Ragin, Michael Markl, James C Carr

**Affiliations:** 1 Radiology, Northwestern University, Carteret, NJ USA; 2 grid.412833.f0000000404676462Radiology, Staten Island University Hospital, New York City, NY USA; 3 grid.465264.7Statistics, Northwestern University, Chicago, IL USA; 4 grid.465264.7Heart Transplant Institute, Northwestern University, Chicago, IL USA; 5 grid.465264.7Cardiology, Northwestern University, Chicago, IL USA

## Background

Endomyocardial biopsy (EMB) is the current gold standard to monitor patients for heart transplant rejection. Quantification of myocardial T2 relaxation time using cardiac magnetic resonance (CMR) has been shown to have high sensitivity and specificity to detect rejection episodes. In this study, we evaluated the prognostic value of myocardial T2 to predict adverse cardiovascular outcomes during 5-year follow-up after initial CMR.

## Methods

49 cardiac transplant patients (mean age, 48 ± 14 years; 65% male) were recruited from a single institution. CMR at 1.5T was performed in all patients, with subsequent quantifications of regional and global myocardial T2. Clinical data was gathered post-CMR over a 5-year period. EMBs were graded according to the International Society of Heart & Lung Transplantation criteria. Rejection episodes were defined by EMB ≥ 2R, new onset heart failure (HF), LV ejection fraction < 40%, or death. Adverse cardiovascular events gathered included coronary artery disease, cardiomyopathies, arrhythmias, and different types of HFs. Multivariable logistic regression and Kaplan-Meier analysis was performed to measure the prognostic value of myocardial T2.

## Results

Myocardial T2 was found to increase with worsening EMB grade from 53.0 ms (0R, no rejection) to 59.9 ms (3R, severe rejection). ROC analysis using two groups (EMBs ≤ 1R and ≥ 2R) showed T2 = 55.0 ms to have the highest specificity and sensitivity. In multivariable analysis that considered T2 measures, age, length of surgery, donor ischemic time, and BMI, both peak T2 (p = 0.038) and septal T2 (p = 0.035) were significant predictors of EMB grade ≥ 2R. Kaplan-Meier analysis showed a significant higher probability of developing EMB ≥ 2R, new onset HF, or death with a global T2 ≥ 55 (p = 0.02). Adverse cardiovascular outcomes of systolic HF, acute systolic HF, and chronic diastolic HF had a higher probability of developing in patients with global T2 ≥ 55 (p = 0.01, p = 0.01, and p < 0.01, respectively).

## Conclusions

CMR T2 quantification demonstrated prognostic value for all-cause rejection and various types of HFs in cardiac transplant patients. These findings suggest that CMR may be a suitable modality to non-invasively monitor cardiac transplant patients for rejection.

**Figure 1:**
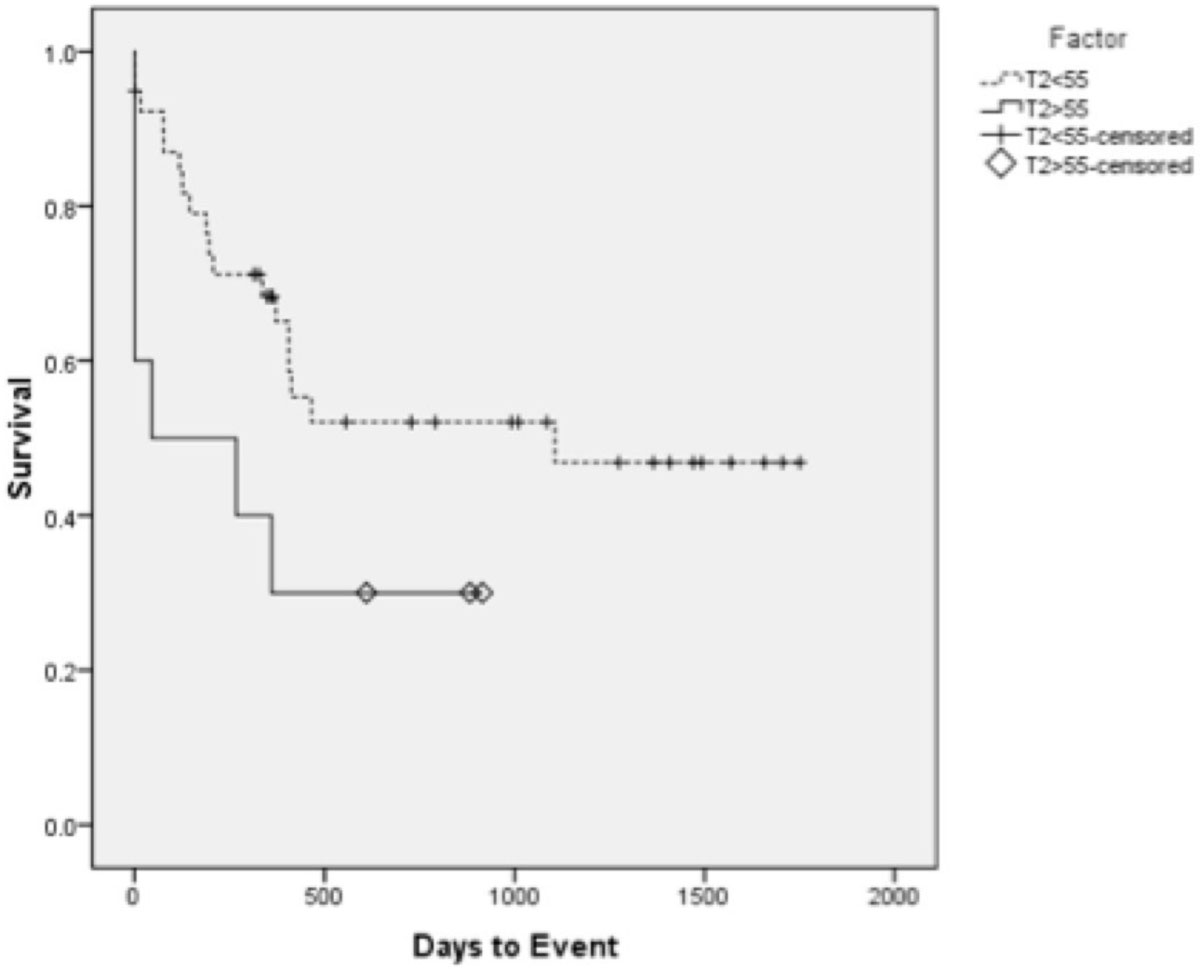
**Kaplan-Meier survival curves for all-cause rejection episodes according to T2 quantification values**. T2 values are dichotomized as ≥55 ms (n = 10, 7 positive outcomes) and <55 ms (n = 38, 18 positive outcomes). Significance level for this composite outcome was 0.02. Factor has units of ms.

**Figure 2:**
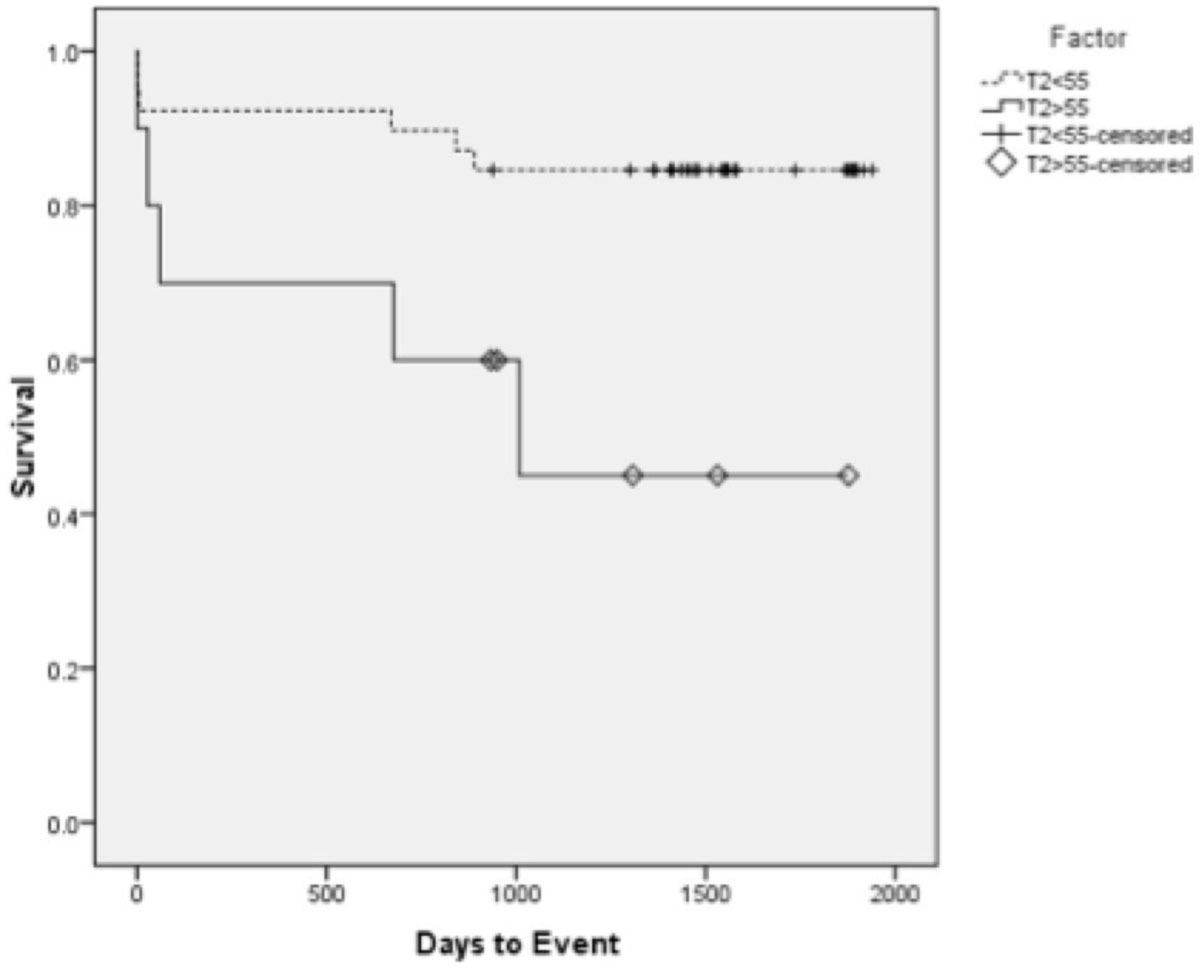
**Kaplan-Meier survival curves for systolic HF episodes according to T2 quantification values**. T2 is dichotomized as ≥55 ms (n = 10, 5 positive outcomes) and <55 ms (n = 39, 6 positive outcomes). Significance level for this composite outcome was 0.01. Factor has units of ms.

